# News and Events of the Week

**Published:** 1910-05-21

**Authors:** 


### News and Events of the Week.

The annual meeting of the Royal British Nurses' Association will be held at 10 Orchard Street, Portman Square, W., on Wednesday, June 15, at 3 p.m.

Mr. Robert Debenham, M.R.C.S., of the Old Rectory, Great Warley, Essex, formerly house surgeon at the London Hospital, who died on March 16, aged seventy-six, left estate of the gross value of £36,862.

Dr. George Skene Keith, M.D., LL.D., F.R.C.P., L.R.C.S., of Moidart Cottage, Currie, Edinburgh, one of the earliest advocates among physicians of the "simple life," who died on January 12, aged ninety-one, left personal estate in the United Kingdom valued at £29,929.

The Samuel D. Gross Prize of the Philadelphia Academy of Surgery for 1910, amounting to $1,500, has been awarded to Astley Paston Cooper Ashhurst, M.D., of Philadelphia, for an essay entitled, "An Anatomical and Surgical Study of Fractures of the Lower End of the Humerus."

The post of Assistant to the Professor of Anatomy and Curator of the Anatomical Museum at University College is vacant through the resignation of Mr. G. S. Hett, while the Sharpey Physiological Research Scholarship is vacant through the resignation of Mr. G. C. M. Mathison, on account of his appointment to a Beit Fellowship.

At the first professional examination in anatomy and physiology for the diploma of Fellow of the Royal College of Surgeons of England, held on May 5, 6, 10, 11, 12, and 13, 135 candidates presented themselves, of whom 73 per cent. were referred back and 27 per cent. were approved. Among the successful candidates is Miss M. M. Basden, of the Royal Free Hospital, who is the first lady to pass this examination, for which six lady students entered.

The Senate of London has received from Professor Karl Pearson a report on the work of the Francis Galton Eugenics Laboratory for 1909-10. On the ground of the national value of its work, Professor Pearson appeals for a more emphatic recognition by the University of the need for full provision for teaching and research in his department. Mr. Heron, Miss Elderton, and Miss Barrington were re-appointed respectively Galton Research Fellow, Scholar, and Computer for another year.

The annual general meeting of the Asylum Workers' Association will be held at 11 Chandos Street, Cavendish Square, W., on Wednesday, May 25, at 3 p.m., Sir William J. Collins, M.D., F.R.C.S., M.P., etc. (President of the Association), in the chair. The annual report will be submitted, officers elected, and presentations will be made of (1) medals for long and meritorious nursing service in asylums, and (2) illuminated address to president and testimonial to hon. secretary in commemoration of passing of Asylum Officers' Superannuation Act. The Rev. Canon Sheppard, D.D., C.V.O. (Sub-Dean of the Chapels Royal), Sir T. Clifford Allbutt, M.D., K.C.B. (Regius Professor of Physic, University of Cambridge), Sir James Moody, Dr. Needham, (Lunacy Commissioner), Professor Bevan-Lewis, M.Sc. (President, Medico-Psychological Association), Mr. J. McD. Henderson, M.P., and Mr. Chas. H. Roberts, M.P., have intimated their hope to be present, in addition to members of the Executive Committee.

A conversazione will be given by the Medical Society of London in the rooms of the Society, 11 Chandos Street, Cavendish Square, W., on Monday, May 23, at 8.30 p.m., when the annual oration will be given by Mr. William H. Battle, F.R.C.S.; subject, "Internal Injuries (Abdominal)."

The *Times* announces that Mr. James Cantlie, the hon. secretary of the Pellagra Commission, has received a telegram from Rome in the following terms: "The Pellagra Field Commission has definitely proved that maize is not the cause of pellagra. The parasitic conveyer is *Similium reptans*.—Sambon."

A bulletin has recently been issued by the U.S. Department of Agriculture dealing with the dispensation of the habit-forming drugs, especially as sold in soothing syrups, asthma cures, and other proprietary medicines advertised as harmless. Photographs of the original packages of some of the more dangerous of these drugs are included in the report, which is said to be the first of a series to be published on this subject by the Department.

Miss Helen Dorothy Smith, of Bedford College, has been appointed the first London University Benington Memorial Student in Craniology and Anthropometry at University College, as from October 1, 1910. The Benington Memorial Studentship was founded this year by Mrs. Norman Robinson, in memory of the late Dr. Robert Crewdson Benington, a former student of University College.

Important new regulations, says the *Child's Guardian*, have been approved by the Home Secretary in relation to street collections in London for charitable and other purposes. One of the most valuable is to the effect that "no boy under the age of fourteen years and no girl under the age of sixteen years shall act as a collector, except at the same place with, and under the charge of, an adult person." It would be a great advantage if this excellent provision was extended to the whole of the country.

The retirement of Mr. Hurry Fenwick from the active surgical staff of the London Hospital, remarks the *Hospital Gazette*, is a serious loss to the hospital and medical school. Mr. Fenwick has justly earned a reputation in that department of surgery with which his name will be ever associated, and his long connection with the "London" has done much to add to its fame as a teaching centre of surgery. Mr. Fenwick has worked assiduously since his appointment to the staff on July 24, 1883. Only those who have worked with him can realise the full value and extent of this work.

A conference of representatives of the City and Metropolitan Borough Councils, convened by the Greenwich Council, will be held at Caxton Hall, Westminster, on the 25th inst., at 3 o'clock, to consider whether it be possible for some authority to maintain a sanatorium for the treatment of metropolitan tuberculous patients, such institution to be supported by a charge upon the several metropolitan boroughs. Numerous metropolitan boards of guardians have written previously to the Metropolitan Asylums Board requesting it to take steps to establish sanatoria for consumptives to be maintained out of the rates.

